# Indoor residual spraying of experimental huts in Cameroon highlights the potential of Fludora® Fusion to control wild pyrethroid-resistant malaria vectors

**DOI:** 10.1186/s12879-024-09630-4

**Published:** 2024-07-25

**Authors:** Riccado F. Thiomela, Magellan Tchouakui, Benjamin D. Menze, Elysee Nchoutpouen, Emilie S. Ngongang-Yipmo, Oliver Wood, Sebastian Horstmann, Raymond J. Mahob, Abraham Fomena, Charles S. Wondji

**Affiliations:** 1 Centre for Research in Infectious Diseases (CRID), P.O. Box 13501, Yaoundé, Cameroon; 2 Department of Animal Biology and Physiology, Faculty of Science, University of Yaoundé 1, P.O. Box 812, Yaoundé, Cameroon; 3 Department of Vector Biology, Liverpool School of Tropical Medicine, Pembroke Place, Liverpool, L35QA UK; 4 International Institute of Tropical Agriculture (IITA), P.O. Box 2008, Yaoundé, Cameroon; 5 Envu 2022 ES Deutschland GmbH, Leverkusen, Deutschland; 6 2022 Environmental Science ZA (Pty) Ltd. (Trading As ENVU), 27 Wrench Road, Kempton Park, 1601 ZA South Africa

**Keywords:** Malaria, Anopheles, Vector control, Indoor residual spraying, Insecticide resistance, Clothianidin, Fludora® Fusion, *Kdr*, *GSTe2*

## Abstract

Elevated resistance to pyrethroids in major malaria vectors has led to the introduction of novel insecticides including neonicotinoids. There is a fear that efficacy of these new insecticides could be impacted by cross-resistance mechanisms from metabolic resistance to pyrethroids. In this study, after evaluating the resistance to deltamethrin, clothianidin and mixture of clothianidin + deltamethrin in the lab using CDC bottle assays, the efficacy of the new IRS formulation Fludora® Fusion was tested in comparison to clothianidin and deltamethrin applied alone using experimental hut trials against wild free-flying pyrethroid-resistant *Anopheles funestus* from Elende and field *An. gambiae* collected from Nkolondom reared in the lab and released in the huts. Additionally, cone tests on the treated walls were performed each month for a period of twelve months to evaluate the residual efficacy of the sprayed products. Furthermore, the L1014F-kdr target-site mutation and the L119F-GSTe2 mediated metabolic resistance to pyrethroids were genotyped on a subset of mosquitoes from the EHT to assess the potential cross-resistance. All Anopheles species tested were fully susceptible to clothianidin and clothianidin + deltamethrin mixture in CDC bottle assay while resistance was noted to deltamethrin. Accordingly, Fludora® Fusion (62.83% vs 42.42%) and clothianidin (64.42% vs 42.42%) induced significantly higher mortality rates in EHT than deltamethrin (42.42%) against free flying *An. funestus* from Elende in month 1 (M1) and no significant difference in mortality was observed between the first (M1) and sixth (M6) months of the evaluation (P > 0.05). However, lower mortality rates were recorded against *An. gambiae* s.s from Nkolondom (mortality rates 50%, 45.56% and 26.68%). *In-situ* cone test on the wall showed a high residual efficacy of Fludora® Fusion and clothianidin on the susceptible strain KISUMU (> 12 months) and moderately on the highly pyrethroid-resistant *An. gambiae* strain from Nkolondom (6 months). Interestingly, no association was observed between the L119F-GSTe2 mutation and the ability of mosquitoes to survive exposure to Fludora® Fusion, whereas a trend was observed with the L1014F-kdr mutation. This study highlights that Fludora® Fusion, through its clothianidin component, has good potential of controlling pyrethroid-resistant mosquitoes with prolonged residual efficacy. This could be therefore an appropriate tool for vector control in several malaria endemic regions.

**Supplementary Information:**

The online version contains supplementary material available at 10.1186/s12879-024-09630-4.

## Background

Vector control interventions including long-lasting insecticide treated nets (LLINs) and indoor residual spraying (IRS) remain among the most effective strategies to prevent malaria transmission [1]. This strategy has significantly contributed to the reduction in malaria cases between 2000 and 2015, with the large-scale deployment of LLINs and IRS averting 68% and 13% malaria cases respectively [2]. Despite the gains achieved by vector control interventions during this period, multiple factors threaten future progress among which resistance of the vectors to the insecticides, and residual transmission. Since 2010, resistance to at least one class of insecticide has been reported in sixty-one countries which not only shortens the lifespan of the existing vector control tools but also undermines the efficacy of novel developed vector control products through cross/multiple resistance [3]. Therefore, the recent gains in reducing malaria transmission could be lost if resistance to insecticides is not well managed.

In response to the challenge of insecticide resistance in malaria vectors, the Global Plan for Insecticide Resistance Management (GPIRM) came up with strategies to preserve the effectiveness of current vector control tools and at the same time develop new and innovative vector control, to significantly reduce malaria morbidity and mortalities [4]. Resistance management rely on the application of insecticides with different biochemical modes of action in IRS and LLINs. These include insecticide rotation, combinations of interventions, mosaic distribution and mixtures of several different insecticides [4]. Among the novel insecticides proposed for managing pyrethroid resistance, neonicotinoids have been presented as a good alternative because they target nicotinic acetylcholine receptors (nAChR) which represent a new biochemical target in public health insects [5, 6]. This insecticide is proposed to be used in rotation as either a mixture (Fludora® Fusion) or in a single formulation (SumiShield) to delay or reverse the spread of resistance [3, 4, 7]. Fludora® Fusion developed by Bayer (Bayer CropScience, Monheim, Germany) is one of the new mixture formulations combining this novel insecticide (clothianidin) and the pyrethroid (deltamethrin) (8:1 w/w) approved for indoor residual spraying (IRS) as a tool for insecticide resistance management (IRM). Field trials using this new formulation demonstrated its high efficacy against various malaria vectors, including pyrethroid-resistant populations of *Anopheles gambiae* [5, 8, 9]. Detecting and monitoring levels of resistance, and understanding how resistance could arise to such new a product is critical to preserve its efficacy.

Resistance to insecticides such as pyrethroid usually arise through target site mutations affecting the voltage gated sodium channel (VGSC, Knock Down Resistance ‘*kdr*’ mutations) and increased insecticide metabolism mediated by detoxification enzymes (metabolic resistance). *Kdr* mutations for example are widely distributed in African *Anopheles* populations and encompass *kdr West* (L1014F) and *kdr East* (L1014S) mutations [6, 7]. Metabolic resistance occurs through increased activities of detoxification enzymes, resulting in increased insecticide metabolism [10]. The main detoxification enzyme families involved include cytochrome P450 monooxygenases (P450, CYP for genes), carboxyl/cholinesterases (CCE), Glutathione-S-transferase (GST), UDP-glycosyl-transferases (UDPGT) and sulfotransferases (SULT). In addition to target-site modifications and metabolic resistance, additional mechanisms involving cuticle modifications, altered insecticide transport and sequestration, sensory appendage protein (SAP) and chemosensory proteins (CSP) have been reported [11–15]. In contrast to pyrethroids, all neonicotinoids act on the insect central nervous system as agonists of the postsynaptic nicotinic acetylcholine receptors (nAChRs). Molecular basis of resistance to this insecticide remains unknow in major malaria vectors despite recent report of clothianidin resistance in *Anopheles funestus* and *An*. g*ambiae* [16–18]. Also, it remains unknown if P450-based or GSTs-based pyrethroid resistance markers could negatively/positively impact the efficacy of neonicotinoid-based control tools such as Fludora® Fusion or SumiShield® 50WG. This should be a critical step before the implementation of clothianidin-based tool in the field. The aim of this study was to evaluate the efficacy and residual effect of Fludora® Fusion compared to clothianidin and deltamethrin applied alone in the field and then determine the impact of known resistance markers on the performance of this new IRS product in field population of *An. gambiae* and *An. funestus* from Cameroon.

### Study area and mosquitoes strain used

**Fig. 1:**
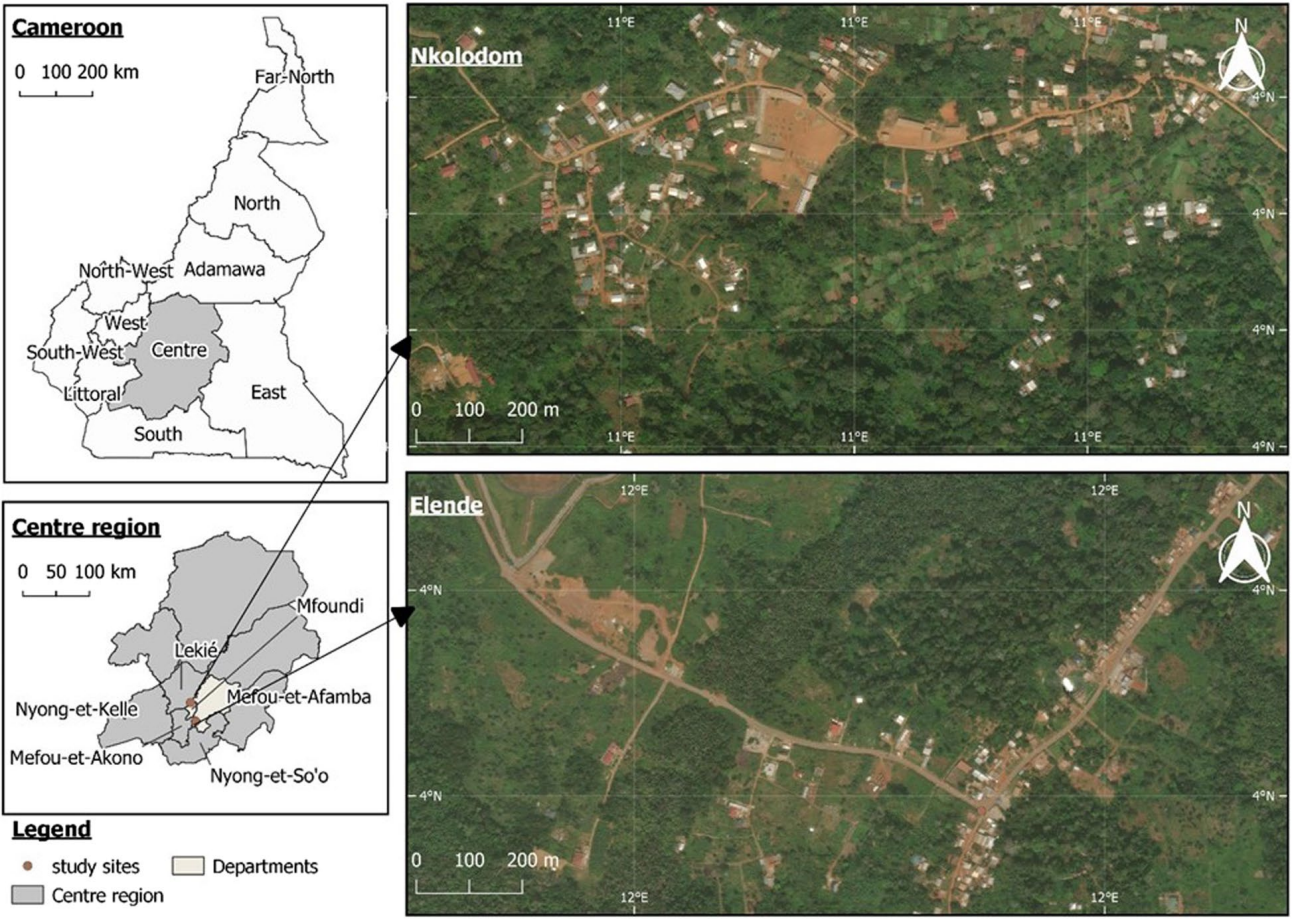
Study and collection site for wild mosquito populations. **A** Nkolondom for Anopheles gambiae s.s. and **B**) Elende for *An. funestus s.s*

The study was performed at the experimental hut station in Elende (3°41′57.27''N, 11°33′28.46''E), a rural village situated in central Cameroon close to Yaoundé (Fig. 1). This village is characterized by a classical Guinean equatorial climate with four distinct seasons: a short rainy season from mid-March to the end of June; a short dry season from late June to mid-August; a long rainy season which runs from mid-August to mid-November and a long dry season which runs from mid-November to mid-March. This locality is highly endemic to malaria mainly driven by *An. funestus s.s*. (792 infective bites/person/year) [19]. Additionally, *An. gambiae* larvae were collected in Nkolondom (3°57′18″ N, 11°29′36″ E), then reared at the CRID insectary and release in the experimental hut. Nkolondom (Fig. 1) is also situated in the Centre region and characterised by extensive agricultural all over the year with massive use of pesticide which have contributed to extremely high level pyrethroids resistance [20, 21] and a recent report of neonicotinoids resistance [22]. Due to the fact that the L1014F mutation is almost fixed in Nkolondom [20], a crossing with the susceptible laboratory strain (Kisumu) was performed to assess the impact of this mutation on the efficacy of IRS products. To perform the crossing, pupae from the susceptible lab strain (kisumu) and the wild population from Nkolondom were collected and put individually into 15 ml falcon tubes for individual emergence. The susceptible females were then mixed in the same cage with the resistant males from Nkolondom for random mating to generate the first generation. The breeding process continued until the second generation, which was used for the bioassays.

### Susceptibility testing in the lab

CDC bottle tests [23] were used to determine the susceptibility of different mosquito populations or strains to the following insecticides:

i. Deltamethrin (DLT): 12.5µg/ml/bottle (acetone as solvent)
ii. Clothianidin (CTD): 90µg/ml/bottle (acetone + mero as solvent)
iii. Fludora.® Fusion (deltamethrin + Clothionidin): 12.5µg/ml/bottle (DLT) + 90µg/ml/bottle (CTD) (acetone + mero as solvent)
iv. Control (acetone)

Sugar-starved adult female Anopheles mosquitoes (20–25), aged 2–5 days, were exposed in the coated bottles (5 replicates for the test + 2 controls for each active ingredient) for 1 h. After exposure, mosquitoes were transferred to resting cups and fed 10% sugar solution for the entire observation period (72 h). Due to the fact that clothianidin is a slow-acting insecticide knock-down was assessed one hour post- exposure and the final mortality assessed at 24, 48 and 72 h.

### Impact of L1014 on the efficacy of Fludora® Fusion

Since the frequency of the L1014F-kdr resistance marker is fixed in the Nkolondom population, the KIS/NKOL hybrid strain resulting from the cross between the Nkolondom wild population and the susceptible lab strain Kisumu were used to determine the effect of this marker on the efficacy of Fludora® Fusion after EHT. The L1014F-kdr marker was genotyped by conventional PCR according to the protocol defined by Martinez-Torres et al. [24].

### Experimental hut trials

#### Experimental hut design

The huts are built according to the West African model recommended by the WHO [25]. They are made of a concrete base surrounded by a drainage channel filled with water to trap ants. The walls are made of concrete bricks and plastered inside and out with a mortar made of a mixture of cement and sand. The roof is made of corrugated iron and the ceiling is made of plywood. The 4 windows located on three sides of the hut are designed from metal pieces attached at an angle to create a funnel with a 1 cm gap, which facilitates the entry of upward flying mosquitoes, but greatly limits their exit once they have entered the hut.

### Experimental hut treatments

The following insecticides were tested in 4 experimental huts:

i. Unsprayed hut (control);
ii. Deltamethrin sprayed at 25 mg/m^2^;
iii. Clothianidin sprayed at 200 mg/m^2^;
iv. Fludora® Fusion sprayed at 25 mg/m^2^ + 200 mg/m^2^.

The huts were sprayed using the *IK vector control* (Goizper Group, Antigua) constant pressure sprayer. To improve the accuracy and quality of the spraying, the various walls and the ceiling were marked in advance with strips and a guide post was attached to the end of the spray wand to maintain a fixed distance from the wall. At the end of the spraying of each hut, the volume of insecticide sprayed was estimated by subtracting the initial volume minus the post-treatment volume.

### Assessment of spraying quality

To ensure that the recommended dose was accurately applied to the substrate, filter papers (Whatman No. 1) measuring 5 × 5 cm were taped to each of four walls and ceiling of each hut. After spraying, the filter papers were removed, carefully wrapped in aluminium foil and stored at 4 ℃ for approximately 2 weeks, after which they were sent to BioGenius GmBH, Germany, for chemical analysis to assess the quality of the spray applications using gas chromatography.

### Hut trial procedure

The trials against free-flying mosquitoes took place over two months in the experimental huts, between September and August 2021 (rainy season) for the first month (M1) and between February and March 2022 (dry season) for the sixth month (M6) post-spraying. The test was conducted according to the protocol described in the WHO guidelines for testing mosquito adulticide for indoor residual spraying and treatment of mosquito nets [26]. Four consenting adult male volunteers slept in the different huts between 20:00 and 06:00 to attract female mosquitoes looking for a blood meal. Sleepers were rotated daily to reduce bias due to individual attractiveness. Each morning, starting at 05:00, mosquitoes were collected in the hut and on the veranda trap using haemolysis tubes. Mosquitoes collected in each compartment were kept in different labelled bags to avoid mixing of samples; they were then transported to the insectary where morphological identification was done and samples stored as live, dead, blood-fed and unfed. Alive mosquitoes were fed with a 10% glucose solution. Mortality was recorded every 24h for 3 days, after which alive specimens were kept in RNA-later and dead ones in silicagel for further molecular analyses. Release-recapture methods were used for mosquitoes coming from the laboratory (crossing Kisumu/Nkolondom (F2)) or from another site (*An. gambiae* from Nkolondom (F0)) than the one where the experimental huts were built. For this approach, the same methodology described above was applied. The difference was that before the releasing, all the openings of the hut were closed, and each test night, 25–30 female mosquitoes aged 5–8 days were released at 8:00 pm and recaptured individually from 5:00 am by the sleepers using haemolysis tubes and stored in bags labelled according to the collection place (veranda or room). The F0 and F2 generations respectively for wild population of An. gambiae from Nkolondom and the Kisumu/Nkolondom cross were used for the trials.

### Outcome measures

The main outcomes measured were:

i. Deterrence effect: reduction in entry into the treated huts relative to the control hut (untreated hut);
ii. Induced exophily: proportion of mosquitoes found in exit traps;
iii. Blood feeding: proportion of blood-fed mosquitoes;
iv. Immediate mortality: proportion of dead mosquitoes at the end of the exposure time;
v. Delayed mortality: proportion of dead mosquitoes after 24, 48, and 72 h.

### Residual efficacy of the IRS products

To assess the residual efficacy of the products on the treated walls, WHO cone bioassays were conducted using 2–5-day-old female mosquitoes of the insecticide susceptible lab strain *An. gambiae* Kisumu and the resistant field population *An. gambiae* from Nkolondom. Bioassays were performed one week after the application of the treatments and a monthly frequency was observed for the first six months while it was quarterly for the last six months. In addition, residual efficacy was assessed at the twelfth month with the wild population of *An. funetus* from Elende. A minimum of 50 mosquitoes of each strain / population were tested per hut in cohorts of 10 per cone on each treated wall/ceiling surface. Mosquitoes were exposed to treated surfaces for 30 min following WHO guidelines [12]. Mortality was recorded 24, 48 and 72 h after exposure.

### Ethical clearance and selection of sleepers

This project was conducted under an ethical Clearance from the National Ethical Clearance Committee of Cameroon No 2021/07/1372/CE/CNERSH/SP. The sleepers were selected from among the local residents after they had read and approved the informed consent describing the study evaluation process in detail. People under the age of 18, women and the sick were excluded. The selected volunteer sleepers were regularly screened for malaria and treated when positive at the village health centre.

### Impact of the L119F-GSTe2 mutation on the effectiveness of different treatments

Samples from the experimental hut trials were grouped into different categories: collection place (veranda, room) and physiological status (alive or dead and blood-fed or unfed). DNA was extracted from each group of sample using the Livak protocol [27]. The L119F-GSTe2 resistance markers was genotyped using the allele-specific PCR on *An. funestus* samples EHT [28] in order to establish its impact on the performance of different treatments.

## Results

### Susceptibility profile of *An. funestus* and *An. gambiae* after CDC bottle assays

**Fig. 2:**
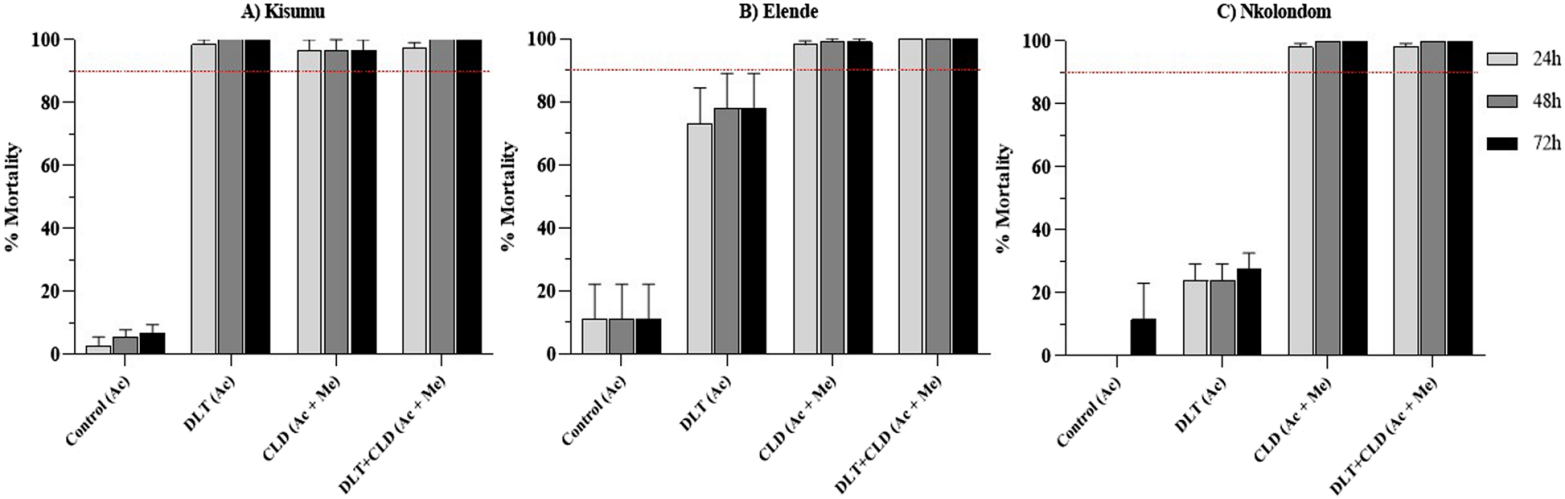
Susceptibility profile of *An. funestus* and *An. gambiae* after CDC bottle assays. Mortality rate of **A**) Kisumu susceptible lab strain, **B**) Anopheles funestus from Elende and **C**) An. gambiae from Nkolondom 24h post exposure to various insecticides; DLT = deltamethrin, CLD = clothianidin, Ac = acetone and Me = Mero

All the populations tested were fully susceptible to clothianidin and Fludora® Fusion (deltamethrin + clothianidin), while the field strains showed resistance to deltamethrin with a mortality rate of 36% for the *An. gambiae* strain from Nkolondom and 78% for the *An. funestus* from Elende (Fig. 2).

### Spray quality control

Assessing the quality of the spray applications using gas chromatography on the filter papers sent to BioGenius GmBH, Germany, for chemical analysis revealed that insecticide concentrations for various treatments was good although some of the deltamethrin walls was slightly overdosed (Table S1)**.**

### Performance of Fludora® Fusion on free flying *Anopheles funestus* from Elende in EHT

A total of 1447 female *Anopheles funestus* mosquitoes were collected during the two months of the evaluation (M1&M6), 630 for the first month and 817 for the sixth month. Several parameters were taken into account for the evaluation of this performance.

### Deterrence effect/entry rate

A significantly higher (*P* < 0.0001) entry rate was observed in the untreated/control hut compared to the treated huts during the first month of evaluation (Table S2). The deterrence effect was higher in huts treated with deltamethrin (80.98%) than in huts treated with clothianidin (70.02%; *P* = 0.1) and Fludora® Fusion (67.4%; *P* = 0.05) although not significant. The hut treated with deltamethrin showed a significantly higher entry rate (45.3%; *P* < 0.0001) compared to other treatments during the sixth month of evaluation (Table S2) indicating a reduced efficacy of deltamethrin six months post treatment.

### Exophily rate

**Fig. 3:**
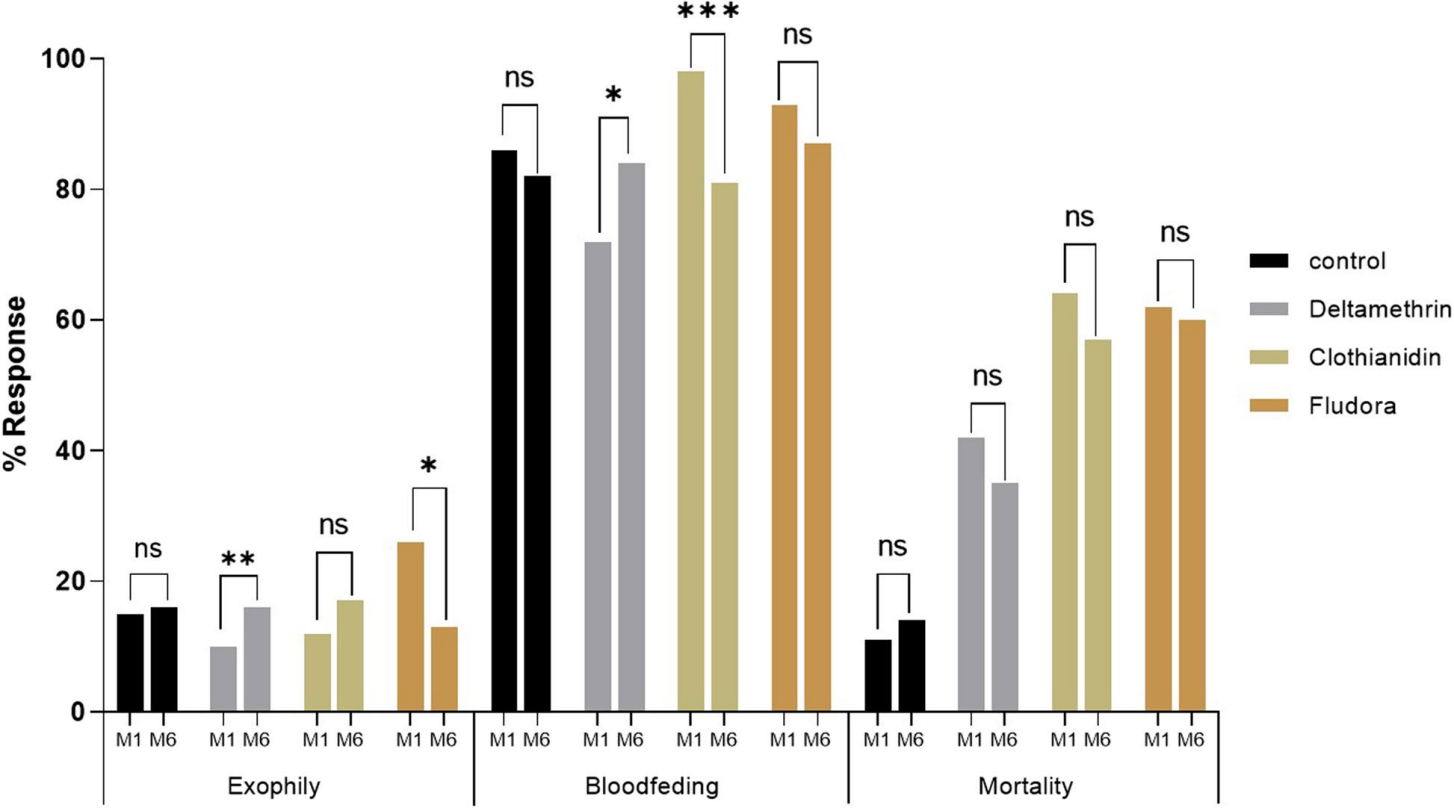
Comparison of IRS product performance between months 1 (M1) and 6 (M6) of the evaluation (ns = no difference, * = *p* < 0.05 ** = *p* < 0.01 *** = *p* < 0.001)

A significantly greater induced exophily was observed in the Fludora® Fusion hut (*P* < 0.01) in month 1 and in deltamethrin hut in month 6 (*P* < 0.01) compared to the other treatments (Table S2). The difference in induced exophily observed between deltamethrin and Fludora® Fusion in month 1 of the evaluation may be related to the low number of mosquitoes collected in the hut treated with deltamethrin (Fig. 3).

### Blood-feeding rate

Due to the absence of a physical barrier with IRS, the blood feeding rate was very high in all treatments (> 72%) (Fig. 3). However, a significantly higher blood feeding rate was observed with clothianidin and Fludora® Fusion in the first month of the evaluation compared to deltamethrin (*P* < 0.05) (Table S2).

### Mortality rate

Mortality rates varied between 11 and 14% in the control hut. This was significantly lower compared to the mortality rates recorded with deltamethrin (42.4%; *P* < 0.01), clothianidin (64.4%; *P* < 0.001) and Fludora® Fusion (62.8%; *P* < 0.001). Mortality recorded in the clothianidin-treated hut was not different to that of Fludora® Fusion treatment (64.42% vs 62.83%; P > 0.05). Also, there was no significant reduction in mortality when comparing the efficacy at months one (M1) and six (M6) for all the treatments (Fig. 3).

### Release-recapture of wild *Anopheles* gambiae from Nkolondom

The results of the performance of IRS products in experimental huts against field pyrethroid-resistant population of *Anopheles gambiae* from Nkolondom are summarised in Table 1. The exophily rate was very high in all treatments including the control. A significantly high induced exophily was observed in the deltamethrin (51.8%; *P* < 0.0001) and Fludora® Fusion (40.7%; *P* < 0.0007) treatments compared to clothianidin (19.3%; *P* < 0.0002). The blood feeding rate was low in all treatments (< 10%) and no significant variation (*P* > 0.05) was observed between the different treatments. In terms of mortality, clothianidin (45.6%; *P* < 0.0001) and Fludora® Fusion (50%; *P* < 0.0001) induced a significantly higher mortality rate than deltamethrin (26.7%) (Table 1).

**Table 1:** Results of the performance of IRS products in experimental huts against pyrethroid-resistant wild *Anopheles gambiae* mosquitoes

	**Control**	**Deltamethrin (25 mg/m2)**	**Clothianidin (200 mg/m2)**	**Fludora® Fusion (225 mg/m2)**
**Females caught**	471	446	450	440
**%Exophily (95% CI)**	29.9 (25.8–34.1)b	51.8 (47.2–56.4)d	19.3 (15.7–23.0)a	40.7 (36.1–45.3)c
**%Blood fed (95% CI**	7.6 (5.2–10.0)a	5.4 (3.3–7.5)a	7.8 (5.3–10.3)a	8.4 (5.8–11.0)a
**% Immediate mortality (95% CI)**	2.3 (1.0–3.7)a	6.1 (3.8–8.3)b	10.4 (7.6–13.3)c	14.3 (11.1–17.6)c
**%Mortality (72 h) (95% CI)**	14.0 (10.9–17.2)a	26.7 (22.6–30.8)b	45.6 (41.0–50.2)c	50 (45.3–54.7)c

Values followed by the same letter along the same line are not significantly different at *P* = 0.05

### Release-recapture of the hybrid Kisumu-Nkolondom *Anopheles* gambiae

The results of the efficacy of different treatments evaluated are summarised in Table 2. Induced exophily was significantly higher in the hut treated with Fludora® Fusion compared to other treatments (Table 2). In comparison to the control, huts treated with deltamethrin and clothianidin had significantly lower blood feeding rates. All treatments induced significantly higher mortality compared to control (*P* < 0.0001). However, clothianidin and Fludora® Fusion induced significantly higher mortality rates than deltamethrin (*P* < 0.01) (Table 2).

**Table 2:** IRS performance against hybrid *An. gambiae* KIS/NKOL

	**Control**	**Deltamethrin (25 mg/m2)**	**Clothianidin (200 mg/m2)**	**Fludora® Fusion (225 mg/m2)**
**Females caught**	221	362	386	411
**%Exophily (95% CI)**	32.6 (26.4–38.8)b	27.5 (22.8–31.9)a,b	24.9 (20.6–29.2)a	51.1 (46.3–55.9)c
**%Blood fed (95% CI**	29.0 (23.0–34.9)b	9.9 (6.9–13.0)a	12.2 (8.9–15.4)a	26.8 (22.5–31.0)b
**% Immediate mortality (95% CI)**	11.8 (7.5–16.0)a	30.7 (25.9–35.4)c	22.5 (18.4–26.7)b	17.8 (14.1–21.5)b
**%Mortality (95% CI)**	22.6 (17.1–28.1)a	54.4 (49.3–59.6)b	83.7 (80.0–87.4)d	69.3 (64.9–73.8)c

Values followed by the same letter along the same line are not significantly different at *P* = 0.05

### Residual efficacy of the IRS products

**Fig. 4:**
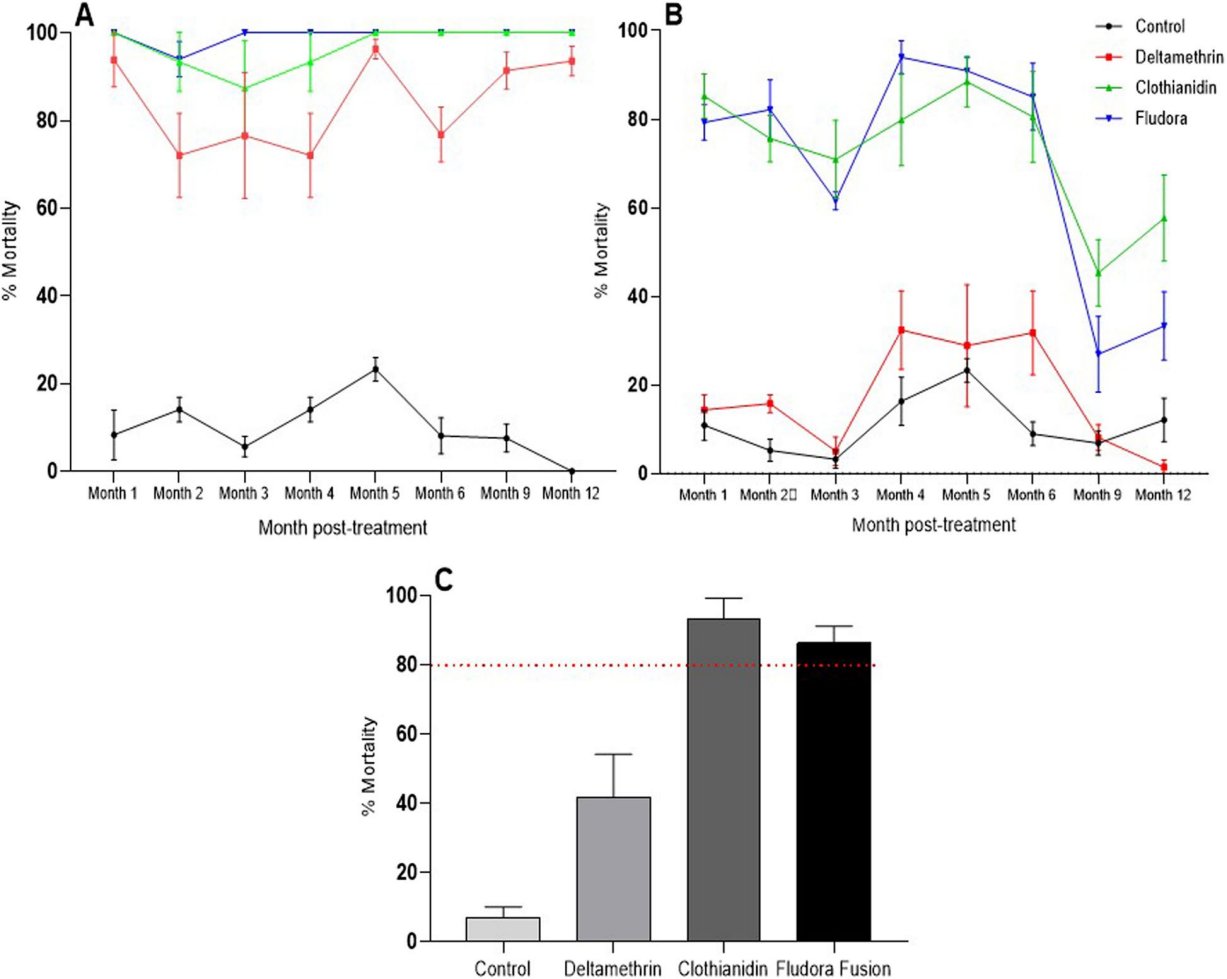
Residual efficacy of the IRS products against the susceptible lab strain Kisumu (**A**) and wild pyrethroid-resistant populations of *Anopheles gambiae* from Nkolondom (**B**) and (**C**) An. funestus from Elende at the twelfth month

Mortality rates obtained after the 30-min cone test on walls with the susceptible laboratory strain KISUMU were, above 80% throughout the evaluation period (12 months), confirming the bioavailability of the active ingredients on treated surfaces (Fig. 4). For the first six months of evaluation, clothianidin and Fludora® Fusion huts induced mortality rates ≥ 80% against the pyrethroid resistant *An. gambiae* strain from Nkolondom except for the third month of evaluation where slight reduction was observed (70% and 62% respectively for clothianidin and Fludora® Fusion). However, mortality rates with deltamethrin did not achieve the 40% level in any case (Fig. 4). The mortality rate significantly reduced from M6 to M12 for all the treatment including Fludora® Fusion and clothianidin indicating a high level of resistance in this field population compared to Kisumu. Additional in-situ cone assay on the wall with *An. funestus* from Elende revealed mortality rates higher than 80% for clothianidin (93.4%) and Fludora® Fusion (86.3%) and 41.67% for deltamethrin after 72h of exposure (Fig. 4).

### Impact of L119F-GSTe2 on the efficacy of Fludora® Fusion

#### Mortality

**Fig. 5:**
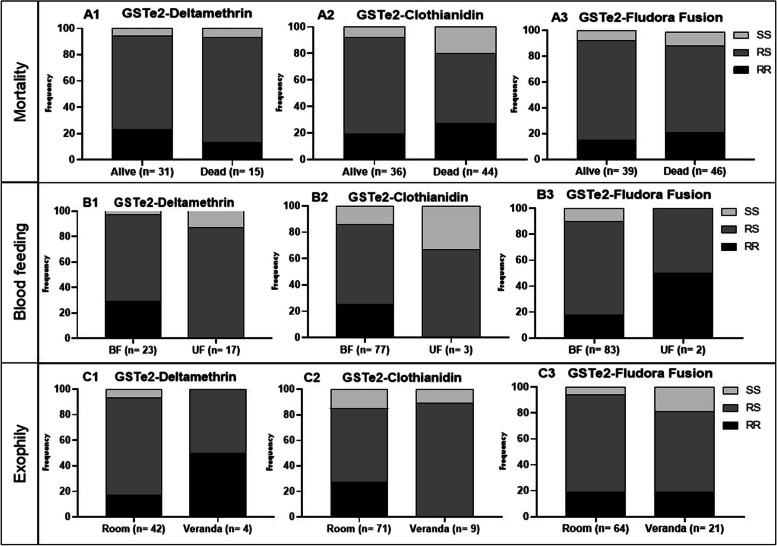
Impact of L119F-GSTe2 mutation on the performance of IRS products in experimental huts against free flying *Anopheles funestus*: Genotype distribution between alive and dead after exposure to deltamethrin (A1), clothianidin (A2) and Fludora® Fusion (A3); genotype distribution between blood fed and unfed after exposure to deltamethrin (B1), clothianidin (B2) and Fludora® Fusion (B3); genotype distribution between indoor (Room) and outdoor (veranda) after exposure to deltamethrin (C1), clothianidin (C2) and Fludora® Fusion (C3). *RR* = *homozygote resistant (119F/F), RS* = *heterozygote (119L/F) and SS* = *homozygote susceptible (119L/L)*

Genotyping of alive and dead mosquitoes after exposure to deltamethrin showed that homozygote resistant have more chance to survive than homozygote susceptible SS (OR = 2.0; P = 0.04) and RS (OR = 1.8; P = 0.3). However, no association was observed for clothianidin and Fludora® Fusion either at the allelic or genotypic level (Fig. 5; Table 3). Due to the low number of unfed mosquitoes (n < 30) and the low number of mosquitoes collected in the veranda trap of the huts of the different treatments, assessment of the impact of the L119F-GSTe2 mutation on the ability to take a blood feeding or to induce exophily was not possible (Fig. 5).

**Table 3:** Impact of L119F-GSTe2 mutation on the efficacy of IRS products in experimental huts against free flying Anopheles funestus

**Mortality**									
	**Deltamethrin**			**Clothianidin**			**Fludora® Fusion**		
**Genotypes**	OR	95% CI	P v	OR	95% CI	P v	OR	95% CI	P v
**RR vs RS**	1.8	0.5 – 6.6	0,3	1.4	0.7– 2.6	0.1	0.6	0.3 – 1.2	0.1
**RR vs SS**	2.0	0.9 – 4.2	0.04	3.6	1.4 – 9.4	0.006	1.07	0.3 – 3.3	0.6
**RS vs SS**	0.8	0.3 – 2.8	0.5	1.4	0.7 – 2.6	0.2	1.6	0.6 –4.8	0.1
**R vs S**	1.3	0.6 – 3.3	0.6	1.1	0.6—2	0.87	0.9	0.5—2	0.88

*OR* odd ratio, *Pv P*-value and *CI* confidence interval

### Impact of the L1014F-Kdr mutation on the efficacy of Fludora® Fusion

#### Mortality

**Fig. 6:**
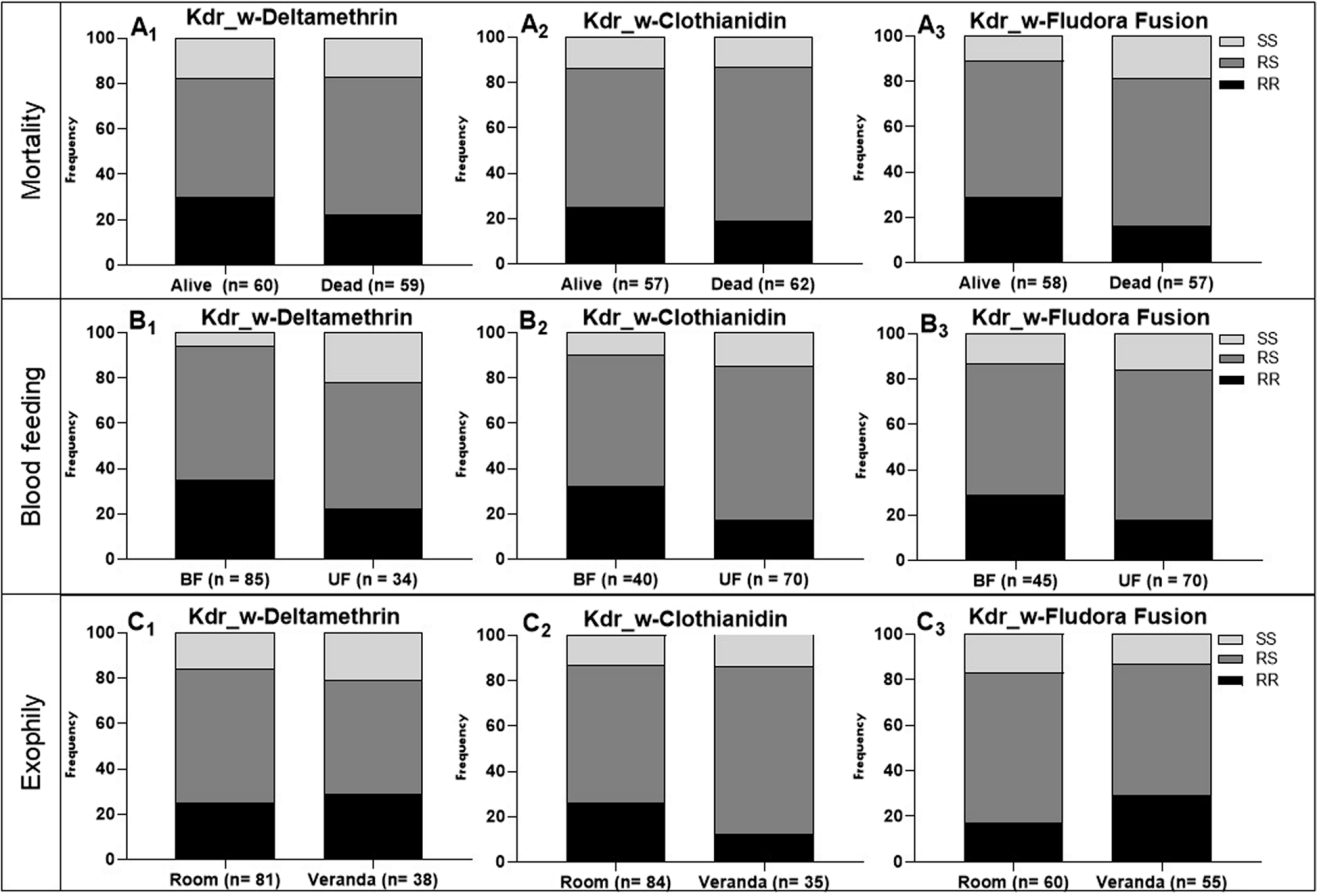
Impact of L1014F-kdr mutation on the performance of IRS products in experimental huts against the hybrid strain Nkolondom/KISUMU (*Anopheles gambiae*): Genotype distribution between alive and dead after exposure to deltamethrin (A1), Clothianidin (A2) and Fludora® Fusion (A3); Genotype distribution between blood fed and unfed after exposure to deltamethrin (B1), Clothianidin (B2) and Fludora® Fusion(B3); Genotype distribution between indoor (Room) and outdoor (veranda) after exposure to (C1) deltamethrin, (C2) Clothianidin and (C3) Fludora® Fusion

Although the homozygote resistant mosquitoes were mainly found among the alive mosquitoes compared to the dead, there was no significant correlation between the presence of the L1014F-Kdr mutation and the ability of mosquitoes to survive exposure to deltamethrin (*χ*^*2*^ = 1; *P* = 0.6) and clothianidin (*χ*^*2*^ = 2; *P* = 0.4). However, whatever the treatment considered, the proportion of RR was higher in the survivors compared to the dead (Fig. 6). In contrast to clothianidin and deltamethrin, with the homozygous resistant genotype (RR) showed a greater ability to survive exposure to Fludora® Fusion compared to heterozygous (RS) (*OR* = 4.4; *P* < 0.05) but no association was observed between RR and SS.

#### Blood feeding

No significant association was found between the presence of the L1014F mutation and the ability of mosquitoes to take a blood meal in huts treated with clothianidin (*χ*^*2*^ = 4.1; *P* = 0.1) and Fludora® Fusion (*χ*^*2*^ = 1.7; *P* = 0.4) (Table 4). It was nevertheless observed that the homozygous resistant genotype had a higher relative frequency in the groups of specimens that had taken a blood meal compared to those that did not (Fig. 6). In contrast to the two previous treatments, a significant association was observed between the L1014F-Kdr resistance allele and an increased ability to take a blood meal in the hut treated with deltamethrin (R vs S; *OR* = 1.8; *P* = 0.04). Comparison of genotypic frequencies showed that RR (RR vs SS; *OR* = 6, *P* = 0.02) and RS (RS vs SS; *OR* = 4, *P* = 0.06) genotypes were more likely to take a blood meal than SS.

**Table 4:** Impact of L1014F Kdr_w mutation on the efficacy of IRS products in experimental huts against the hybrid strain Nkolondom/KISUMU (*Anopheles gambiae*)

**Mortality**									
	**Deltamethrin**			**Clothianidin**			**Fludora® Fusion**		
**Genotypes**	**OR**	**95% CI**	**P v**	**OR**	**95% CI**	**P v**	**OR**	**95% CI**	**P v**
**RR vs RS**	1.7	0.6—4.8	0.4	1.8	0.6—4.8	0.3	4.4	1.1—18.1	**0.04**
**RR vs SS**	1.2	0.3—4.7	1	0.8	0.2—3.7	1	6	1—35.9	0.1
**RS vs SS**	0.7	0.2—2.4	0.8	0.5	0.1—1.8	0.3	1.4	0.3—5.6	0.7
**R vs S**	1.2	0.6—2.2	0.6	1	0.6—1.9	1	2	1—3.9	0.1
**Blood feeding**									
**RR vs RS**	1.5	0.6—3.6	0.4	2.4	0.9—5.8	0.1	1.7	0.7—4.4	0.2
**RR vs SS**	6	1.2—30.5	0.02	3	0.8—11.8	0.1	1.8	0.5—6.5	0.3
**RS vs SS**	4	0.9—19	0.1	1.3	0.4—4.4	0.8	1	0.3—3.1	1
**R vs S**	1.8	1—3.3	0. 04	1.5	0.9—2.7	0.1	1.3	0.8—2.2	0.4
**Exophily**									
**RR vs RS**	1.4	0.6—3.4	0.5	0.4	0.1—1.1	0.1	2	0.8 – 5	0.1
**RR vs SS**	0.9	0.3—2.8	0.8	0.4	0.1—1.8	0.3	2.3	0.7 – 8	0.2
**RS vs SS**	0.6	0.2—1.8	0.4	1.1	0.4—3.6	0.8	1.1	0.4—3.3	0.8
**R vs S**	1	0.6—1.7	1	0.7	0.4—1.3	0.3	1.4	0.8—2.3	0.2

*OR* odd ratio, *Pv P*-value and *CI* confidence interval

#### Exophily

As observed with the previous parameters, no significant correlation was established between the presence of the L1014F-kdrw mutation and the induced exophily, whatever the treatment (Table 4). However, it was observed that, contrary to clothianidin, the proportion of specimens of RR genotypes collected in the veranda trap was higher than that collected in the room of deltamethrin and Fludora® Fusion treatments (Fig. 6).

## Discussion

Aggravation of pyrethroid resistance in the major malaria vectors presents a serious threat to vector control interventions in Africa and has led to the introduction of new insecticides, such as neonicotinoids. To prolong the effectiveness of these new products, it is vital to keep vigil on potential cross-resistance conferred by metabolic resistance to pyrethroids. The present study evaluated the ability of neonicotinoid-based IRS formulation Fludora® Fusion to control pyrethroid-resistant malaria vectors in laboratory and experimental hut in Cameroon and assessed the impact of known molecular markers on its efficacy.

### Combination of deltamethrin with clothianidin induced significantly higher mortality in CDC  bottle assays compared to deltamethrin alone

Full susceptibility of all populations/strains tested to the deltamethrin/clothianidin mixture was observed using CDC bottle bioassays, while wild populations of Nkolondom (*An. gambiae*) and Elende (*An. funestus*) were resistant to deltamethrin. This susceptibility to the clothianidin/deltamethrin mixture is mainly due to clothianidin which targets nicotinic acetylcholine receptors and previous studies reported that malaria vectors across the continent are broadly susceptible to the insecticide when using the appropriate solvent (acetone + MERO) [16, 17]. Nevertheless, it was also reported that MERO could mask resistance to clothianidin as reduced susceptibility was observed in some location where absolute ethanol or acetone alone was used as solvents [16, 22]. Resistance to deltamethrin obtained here are confirmed by previous work carried out in the same sites showing extreme level of pyrethroid resistance but susceptibility to clothianidin [20, 29]. This indicates the need to introduce new intervention tools using new non-pyrethroid active molecules, such as clothianidin, to combat vectors on the continent.

### Fludora® Fusion showed very high performance on *An. funestus* and *An. gambiae* in the EHT compared to deltamethrin

Fludora® Fusion induced the higher mortality in all the populations tested compared to deltamethrin. As stated above, the higher efficacy of Fludora® Fusion is associated to the clothianidin component as previously reported in other countries [8, 9, 30, 31]. The low mortality response recorded with deltamethrin IRS in the experimental huts is similar to the recent observations in Benin [5, 8] pointing to the urgent need of alternative control tools for IRS interventions. Our study demonstrates for the first time the efficacy of Fludora® Fusion against wild free-flying pyrethroid-resistant malaria vectors in semi-field condition in Cameroon. Similar efficacy of this new IRS formulation was previously reported in other African countries [8, 9, 30–32] confirming the suitability of Fludora® Fusion for indoor residual spraying in Cameroon and other malaria-endemic areas where resistance has escalated to pyrethroid. However a lower mortality rate was observed with the *An. gambiae* population from Nkolondom where neonicotinoid resistance has been previously reported [22] compared to the *An. funestus* population (61.79% vs 50%) from Elende. The reduced efficacy of Fludora® Fusion against the *Anopheles gambiae* population from Nkolondom could be associated to the emergence of resistance due to pre-exposure to residues of neonicotinoid insecticides in breeding sites in this agricultural setting [33]. Farmers in Nkolondom use several neonicotinoid-based pesticides such as Benji® (active ingredient = acetamiprid), Optimal (active ingredient = clothianidin), to protect crops from pests. This pesticide usage could easily pollute larval habitats and induce the expression of protective mechanisms in vectors as previously reported that pre-exposure in the field to imidacloprid and acetamiprid induces cross-resistance to clothianidin and reduces the efficacy of the clothianidin-based tools SumiShield® 50WG [33]. All this shows that Fludora® Fusion could rapidly lose the efficacy in areas of intense agricultural practices if not well managed. In all experiments, Clothianidin alone induced higher mortality than Fludora® Fusion. Using hybrid strain from the crossing between the resistant field population (from Nkolondom) and the susceptible laboratory strain (Kisumu), clothianidin sprayed alone induced significantly higher mortality than Fludora® Fusion. This difference in mortality observed could, on the one hand, be linked to the high exophily induced by Fludora® Fusion due to the irritant effect of its deltamethrin component, which prevents mosquitoes from resting long enough on the walls of treated huts to absorb the lethal dose of active ingredients and, secondly, to the difference in the physiological protection mechanism brought into play by the specimens after exposure to clothianidin alone and to the clothianidin plus deltamethrin mixture, as previously reported by Zoh et al. [18]. Similarly, previous studies in experimental huts have also shown a significant reduction in mortality with the chlorfenapyr + alpha-cypermethrin IRS mixture compared with chlorfenapyr alone, due to the irritant effect of the alpha-cypermethrin contained in the mixture [34].

### Fludora® Fusion did not induce high blood feeding inhibition

The blood-feeding rates obtained during the evaluation in the experimental hut with free-flying *Anopheles funestus* were very high in all treatments (generally over 80%). Unlike LLINs, IRS is not a physical barrier that limits blood feeding, but a chemical trap that targets blood-feeding vectors that rest on sprayed surfaces and absorb a lethal dose of insecticide. Similar blood feeding patterns have been obtained in several other countries and even with different classes of insecticide [5, 8, 34, 35]. As previously obtained in Benin [8] Fludora® Fusion did not induce any inhibition of blood feeding in this study compared with the control irrespective of the population or strain tested. This indicate that additional intervention such as bed nets or house improvement should be combined with Fludora® Fusion to guarantee a high personal protection rate to the populations as mosquitoes could continue transmitting the disease before been killed by the insecticide.

### Fludora® Fusion compared to clothianidin induced more exophily on both *An. funestus* and *An. gambiae*

For all the populations tested, mosquito exiting rates were higher in the huts treated with deltamethrin and Fludora® Fusion than in those treated with clothianidin alone and control. This excito-repellent effect observed with Fludora® Fusion is undeniably associated with the deltamethrin contained in the mixture as previously reported in Benin [8]. This effect of pyrethroids had been also reported in the past evaluation of the efficacy of mosquito nets in experimental huts in Cameroon [36]. This study showed a variation in the excito-repellent effect linked not only to the class of insecticide sprayed but also to the species tested; this excito-repellent effect was significantly higher with *Anopheles gambiae* populations compared to *An. funestus*. The exophily induced by Fludora® Fusion was significantly higher (*P* < 0.05) with *Anopheles gambiae* (41%) compared to *An. funestus* (26%) which could be associated to the fact that *Anopheles gambiae* has a greater tendency to exophily/exophagous behaviour than *Anopheles funestus* as reported by entomological studies [19]. This exophilic tendency reduces the contact time of the vectors with the sprayed surfaces and consequently the amount of active ingredient absorbed. This would explain the lower mortality observed in *An. gambiae* compared to *An. funestus*. Overall, the higher insecticide induced exiting rate observed with Fludora® Fusion compared to clothianidin alone in this study and others [8, 30, 31] is important for reducing indoor resting and biting which may contribute to lowering transmission intensities.

### Fludora® Fusion displayed a higher residual effect on both *An. funestus* and *An. gambiae* compared to deltamethrin

The results of the WHO cone bioassays on the treated surfaces of the experimental huts amply demonstrate the ability of Fludora® Fusion to induce high levels of mortality (> 80%) in pyrethroid-resistant mosquitoes; with efficacy prolonged for up to 6 and 12 months against wild populations of *An. gambiae* and *An. funestus* respectively after spraying. Deltamethrin applied alone showed low residual activity against pyrethroid-resistant wild populations, with mortality rates below 50% throughout the evaluation period. In view of the above, Fludora® Fusion could be a good candidate for IRS in Cameroon, as its residual activity covers the period of high malaria transmission in all parts of the country. This prolonged residual activity could be explained by the low volatility of clothianidin supported by its very low vapour pressure (9.8*10^–10^ mmHg at 25°C), which significantly reduces the risk of loss of the active ingredient by evaporation into the atmosphere and ensures its availability on sprayed surfaces for a long period of time [37]. Similar residual efficacy has been reported in several countries where malaria is endemic and vectors are resistant to pyrethroids [5, 8, 9, 30, 31].

### The L1014F-kdr pyrethroid resistance marker was associated with resistance to Fludora® Fusion in the EHT whereas the L119F-GSTe2 mutation had no impact

A positive association was found between the modification of the target site (L1014F-Kdr) in *Anopheles gambiae* and the ability to survive exposure to Fludora® Fusion with mosquitoes harbouring the L1014F mutation more able to survive in the presence of Fludora® Fusion compared to their susceptible counterparts. This could be due to deltamethrin component in this IRS product as reported that the L1014F mutation is strongly associated with deltamethrin resistance across the continent [24, 38]. However, no association was observed between this marker and deltamethrin resistance in this study showing that the impact could be different in an IRS product compared to standard WHO tube or CDC bottle assays. Similarly, a negative association was obtained by Tchouakui et al*.* between the L1014F mutation and the ability to survive exposure to clothianidin in a CDC bottle test [16] whereas no association was observed in this study after EHT. Furthermore**,** no association was found between the DDT/pyrethroid resistance marker L119F-GSTe2 and the ability of *Anopheles funestus* to survive exposure to Fludora® Fusion whereas recent studies show a strong association between L119F-GSTe2 and the ability to survive exposure to clothianidin with CDC bottle test [17]. The difference could be due to the method used as experimental hut trial takes into consideration the behaviour of the mosquitoes, which is not the case for tests in bottles, which is a direct mortality exposure test. All this shows that there is less chance of cross-resistance between *GSTe2* and clothianidin based-IRS whereas *kdr* mutation could comprise the efficacy of these tools.

## Conclusion

This study shows that Fludora® Fusion, is effective in controlling pyrethroid-resistant malaria vectors in Cameroon, as previously shown in many other African countries. However, reduced efficacy of this dual AI IRS product was noticed on kdr-resistant *An. gambiae* compared to *An. funestus* which were more susceptible. Interestingly, Fludora® Fusion had prolonged residual activity ranging from 6 months against *An. gambiae* to 12 months against *An. funestus* showing that this could be therefore an appropriate tool for vector control in several malaria endemic regions.

### Supplementary Information

Supplementary Material 1.

Supplementary Material 2.

## Data Availability

Data is provided within the manuscript or supplementary information files.

## Abbreviations

CDC: Center for Disease Control and Preventions
IRS: Indoor Residual Spraying
EHT: Experimental Hut trial
LLIN: Long-Lasting Insecticidal Net
GPIRM: Global Plan for Insecticide Resistance Management
IRM: Insecticide Resistance Management
DLT: Deltamethrin
CTD: Clothianidin
WHO: World Health Organisation
DNA: DeoxyriboNucleic Acid
PCR: Polymerase chain reaction
RR: Homozygote resistant
RS: Heterozygote
SS: Homozygote susceptible
