# Supplementary material for: Indoor residual spraying of experimental huts in Cameroon highlights the potential of Fludora® Fusion to control wild pyrethroid-resistant malaria vectors
Source: BMC Infect Dis. 2024 Jul 25;24:733. doi: 10.1186/s12879-024-09630-4 (PMC11271210; doi:10.1186/s12879-024-09630-4)
Supplement: Supplementary file 2 — Supplementary Material 2. [file 12879_2024_9630_MOESM2_ESM.docx]

**Table S2**: Results of the performance of IRS products in experimental hut trials against free-flying pyrethroid-resistant *Anopheles funestus*.

| **MONTH 1** | | | | |
| --- | --- | --- | --- | --- |
|  | **Control** | **Deltamethrin (25 mg/m2)** | **Clothianidin (200 mg/m2)** | **Fludora® Fusion (200 mg/m2)** |
| **Females caught** | 347 | 66 | 104 | 113 |
| **Entry rate** | 55.08 a | 10.48 b | 16.51 b | 17.94 b |
| **%Exophily (95% CI)** | 15.20 (11.49-19.06) a | 10.61 (3.18-18.03) a | 12.50 (6.14-18.86) a | 26.55 (18.41-34.69) b |
| **%Blood fed (95% CI)** | 86.46 (82.85-90.06) b | 72.73 (61.98-83.47) a | 98.08 (95.44-100.72) c | 93.81 (89.36-98.25) c |
| **%Mortality (72h) (95% CI)** | 11.52 (8.17-14.89) a | 42.42 (30.50-54.35) b | 64.42 (55.22-73.62) c | 62.83 (53.92-71.74) c |
| **Deterrence** | 0 | 80.98 | 70.02 | 67.43 |
| **MONTH 2** | | | | |
| **Females caught** | 167 | 362 | 173 | 107 |
| **Entry rate** | 20.44 b | 45.29 a | 21.18 b | 13.10 b |
| **%Exophily (95% CI)** | 16.17 (10.58-21.75) a | 25.41 (20.93-29.90) b | 17.34 (11.70-22.98) a | 13.08 (6.69-19.47) a |
| **%Blood fed (95% CI)** | 82.63 (76.89-88.38) a | 84.25 (80.50-88.01) a | 81.50 (75.72-87.29) a | 87.85 (81.66-94.04) a |
| **%Mortality (72h) (95% CI)** | 14.37 (9.05-19.69) a | 36.74 (31.77-41.71) b | 57.80 (50.44-65.16) c | 60.75 (51.50-70.00) c |
| **Deterrence** | 0 | -121.56 | -3.59 | 35.93 |

(Values followed by the same letter along the same line are not significantly different at the 5% threshold)
